# Knockout tales: the versatile roles of histone H3.3 in development and disease

**DOI:** 10.1186/s13072-023-00512-8

**Published:** 2023-10-10

**Authors:** Rachel H. Klein, Paul S. Knoepfler

**Affiliations:** 1 Department of Cell Biology and Human Anatomy, University of California Davis, Davis, CA 95616 USA; 2 Institute for Pediatric Regenerative Medicine, Shriners Hospital for Children Northern California, Sacramento, CA 95817 USA; 3 Genome Center, University of California Davis, Davis, CA 95616 USA

**Keywords:** Histone H3.3, H3f3a, H3f3b, Mouse knockouts

## Abstract

Histone variant H3.3 plays novel roles in development as compared to canonical H3 proteins and is the most commonly mutated histone protein of any kind in human disease. Here we discuss how gene targeting studies of the two H3.3-coding genes *H3f3a* and *H3f3b* have provided important insights into H3.3 functions including in gametes as well as brain and lung development. Knockouts have also provided insights into the important roles of H3.3 in maintaining genomic stability and chromatin organization, processes that are also affected when H3.3 is mutated in human diseases such as pediatric tumors and neurodevelopmental syndromes. Overall, H3.3 is a unique histone linking development and disease via epigenomic machinery.

## Background

DNA is wrapped around an octamer of histones constituting the nucleosome, which helps to organize and compact the genetic material [1]. A substantial amount of gene regulation occurs at the level of these histones. For example, post-translational modifications (PTMs) of amino acid residues within their N-terminal tails influence the recruitment of transcription factors and other chromatin regulators, as well as the level of compaction of the chromatin at a given gene locus [2–4]. Additionally, multiple histone variants exist and show preferential enrichment at different classes of functional domains, further adding to the complexity of this form of epigenetic regulation [5].

Histone H3.3 is an example of a histone variant with a number of unique properties. Unlike canonical histones, which occur in gene clusters, H3.3 is encoded by just two genes, *H3f3a* and *H3f3b*. These two genes also differ from canonical histone genes in that they contain introns and their mRNA is polyadenylated. In contrast to the coordinated gene expression that occurs in histone gene clusters, *H3f3a* and *H3f3b* each have unique untranslated regions, promoters, and expression patterns [6–8].

Direct comparisons to canonical H3 proteins reveal that H3.3 differs from H3.1 and H3.2 at 5 and 4 amino acid residues, respectively. Residues that are fully unique to H3.3 are S31, A87, I89, and G90. Phosphorylation of the unique S31 of H3.3 occurs in a number of regulatory contexts and during mitosis [9], while residues 87, 89, and 90 play a role in another important property of H3.3: unlike canonical H3.1 or H3.2, H3.3 can be deposited on chromatin in a replication-independent manner through unique interactions with chaperones HIRA or ATRX and DAXX [10–12]. While replication-independent deposition is an important function of H3.3 in all cells, it takes on a potentially outsized importance in post-mitotic cells, where H3.3 can still be deposited in chromatin, often progressively replacing canonical H3 in nucleosomes over time [13]. Phosphorylation of S31 has several functional roles including at enhancers [14].

Histone variant H3.3 is one of the most highly conserved proteins across eukaryotic species [15]. In fact, the protein sequence is identical in all vertebrates examined. Despite identical amino acid sequences in the H3.3 protein encoded by *H3f3a* and *H3f3b*, codon usage differs between the two genes and appears to be under purifying selection [16]. Intriguingly, *H3f3a* codon usage is more closely aligned with codon usage in proliferation-associated genes, whereas *H3f3b* codon usage is more comparable to differentiation-associated genes [16], suggesting that over the course of evolution *H3f3a* and *H3f3b* may have become optimized for unique transcriptional programs.

H3.3 has a complex role in gene regulation; it is associated with active regions and open chromatin, but is also found at telomeres and repressed genes [11], suggesting context-specific functions in gene regulation and chromosome stability. Despite encoding identical protein sequences, *H3f3a* and *H3f3b* appear to have some unique and non-overlapping roles in gene regulation.

Many challenges exist to unraveling the shared and unique roles of histone H3 family members through knockout approaches. These include the fact that multiple variants exist: histone H3 has 5 variants. In addition, in the case of canonical H3 histones like H3.1 and H3.2, there are multiple individual genes arranged in gene clusters to allow for the high level of histone expression needed during S phase [17]. Additionally, many redundancies exist between the different histone H3 variants. Histone proteins are also long-lived, with turnover rates sometimes measured in weeks rather than hours [18–20].

**Fig. 1:**
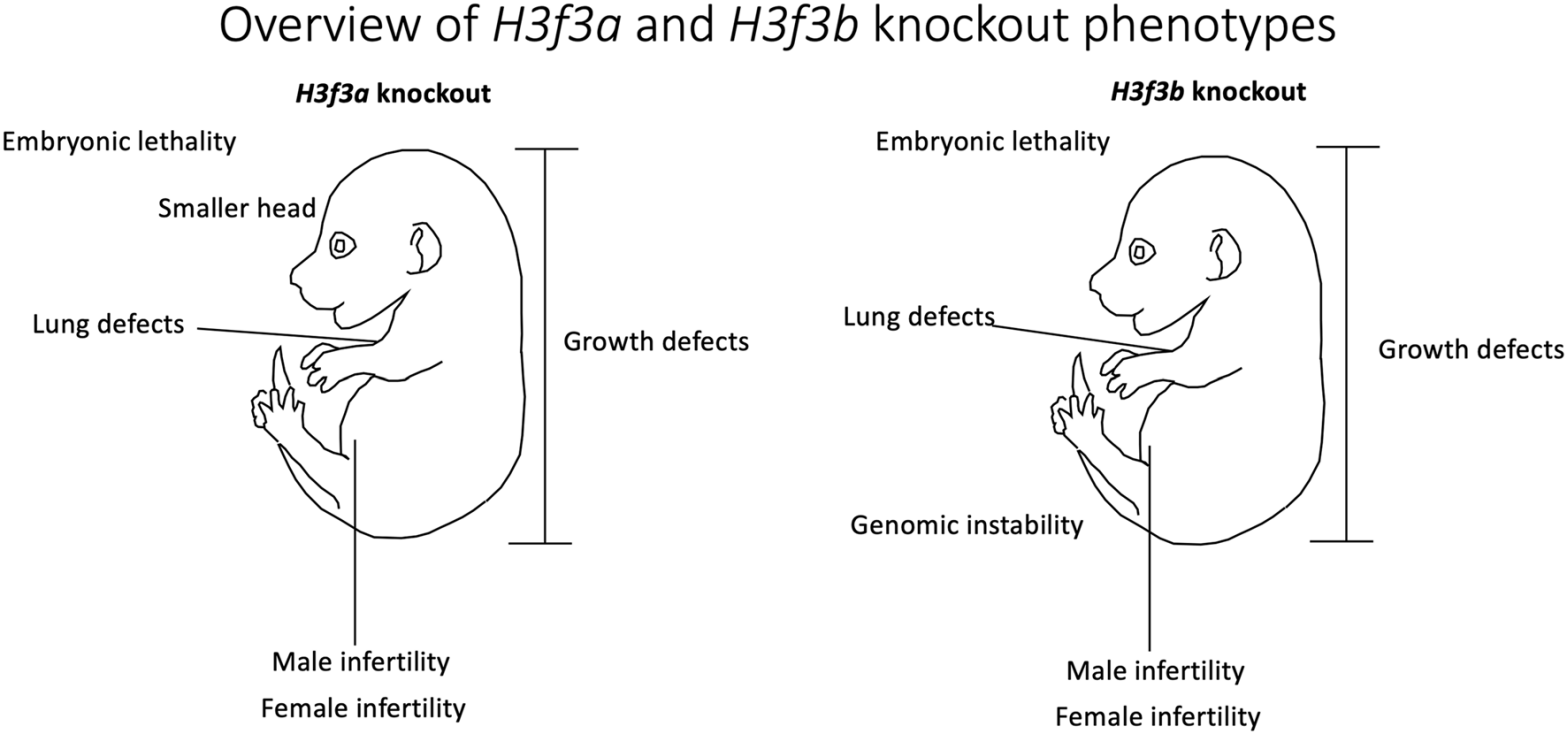
Overview of *H3f3a* and *H3f3b* knockout phenotypes. Mouse embryo image in diagram was adapted from a Theiler stage reference diagram

Despite these challenges, several knockout and loss-of-function studies have shed light on the important and distinct roles of *H3f3a* and *H3f3b* during development (Table 1). One of the most striking findings was the effect of loss of *H3f3a* or *H3f3b* on fertility and gamete formation (Fig. 1). It appears that both genes have vital and non-redundant roles in gamete formation in males, and that *H3f3b* is also required to varying degrees for gamete formation in females [21–23]. In many other developmental processes, knockout studies indicated that *H3f3a* and *H3f3b* were able to compensate for each other, however loss of both resulted in severe growth defects due to genomic instability and activation of the p53 pathway [24]. This role in maintaining the genome appears to be more important than specific gene regulation, as gene expression studies have repeatedly uncovered only modest changes in gene expression with loss of one or both H3.3 genes [22, 25].

**Table 1:** Published H3.3 single knockouts and their phenotypes

Gene knockout	Knockout phenotype	References
*H3f3a*, gene trap	50% neonatal mortality, neuromuscular deficits, reduced fertility in males and females	Couldrey et al. [26]
*H3f3a*, Cre-LoxP	Reduced size, male infertility due to reduced sperm motility and head/tail defects	Tang et al. [23]
*H3f3a,* knockout-first	Almost full embryonic lethality, smaller heads, underdeveloped lungs, some growth defects in heterozygotes	Bush et al. [27]
*H3f3b,* Cre-LoxP (Zp3-Cre)	Partial embryonic lethality, abnormal embryonic development, infertility	Yuen et al. [21]
*H3f3b,* Cre-LoxP (Hprt1-Cre)	Full embryonic/perinatal mortality, growth defects and male infertility in heterozygotes	Tang et al. [23]

## Knockout of *H3f3a*

The first data on knockout of an H3.3 gene in mice came from a gene trap experiment aimed at identifying genes involved in spermatogenesis. Using a LacZ reporter construct, *H3f3a* was identified as a gene with high expression in the testes [26]. Further characterization revealed high expression in both male and female gonads, specifically in primary spermatocytes and in developing eggs [26].

The gene trap approach resulted in a very strong reduction in *H3f3a*; levels were below detection by Northern blot, but some *H3f3a* could still be detected by RT-qPCR. Fifty percent of these homozygous *H3f3a* mutant pups died shortly after birth, and those that survived showed reduced abilities to compete with their wild-type (WT) littermates, likely due to neuromuscular deficits, as standardized behavior screens showed significant differences between WT and *H3f3a* mutant mice [26]. Fertility was also found to be reduced in both male and female *H3f3a* mutant mice, despite reproductive organs appearing normal [26].

Surprisingly, knockout of *H3f3a* using a Cre-LoxP approach in 129S1/SvImJ (129S1) mice, did not result in the peri-natal lethality observed using the gene trap approach [23]. These *H3f3a* knockout mice appeared phenotypically normal at birth and survived to adulthood in normal Mendelian ratios, although *H3f3a*^*−*/−^ males were smaller than WT littermates at 3 and 6-week timepoints, suggesting a mild potential growth delay [23].

In this study, while *H3f3a*^*−/−*^ females had normal fertility, *H3f3a*^*−/−*^ males showed signs of reduced fertility and sired smaller litters than WT males [23]. Closer examination revealed lower levels of fertilized ova from the sperm of *H3f3a*^*−/−*^ males, likely due to reduced sperm motility as well as head and tail defects [23]. These results indicate that *H3f3a* is required for spermatogenesis and has a non-redundant role with *H3f3b* in this process.

A recent paper using a knockout-first approach to disruption of *H3f3a* observed an even more pronounced phenotype, with almost full embryonic lethality in homozygous null embryos, as well as growth delay in approximately 50% of the heterozygotes [27]. *H3f3a* knockout embryos had smaller heads and underdeveloped lungs, indicating defects in key developmental processes in the absence of *H3f3a*.

The finding that knockout of *H3f3a* resulted in distinct phenotypes in different studies was unexpected: half of pups died perinatally in one study, while another study showed nearly full embryonic lethality, and a third study found no defects in viability. One potential confounding variable that could lead to differences in phenotypes is the longevity of the H3.3 protein itself: even after genetic deletion, H3.3 protein will continue to be present in developing cells for a long period of time, which may influence the severity of the ultimate phenotype. Observed differences in phenotype may also be the result of different background strains of mice used in the various studies or may have to do with the efficiency of the depletion.

The gene-trap approach resulted in very low levels of *H3f3a* but was not a full knockout [26]. The Cre-LoxP approach to *H3f3a* knockout used in the second study resulted in excision of the floxed sequence shortly after fertilization of Cre-containing oocytes, and the authors reported very clean deletion with no mosaicism in the resulting offspring [23, 28]. The third study with a knockout first approach that includes the insertion of a large cassette to disrupt the *H3f3a* locus also resulted in very low but detectable levels of *H3f3a* mRNA by qPCR and also observed a more severe phenotype [27]. It is a surprising, but never the less a consistent finding, that the two approaches with residual levels of *H3f3a* mRNA also showed more severe phenotypes than the full Cre deletion. It is possible that insertion of genetic material into the *H3f3a* locus in the gene trap and knockout-first approaches has a different, and more deleterious effect, than a Cre-based deletion of the locus.

## Knockout of *H3f3b*

The effects of *H3f3b* loss in mice was studied using a Cre-LoxP system of knockout. *H3f3b*-floxed mice were crossed with Zp3-Cre to create a germline deletion of *H3f3b*. Heterozygous (*H3f3b*^*+/−*^) mice were recovered in litters at slightly lower than expected ratios, which may indicate a low level of lethality, while *H3f3b*^*−/−*^ mice were recovered at much lower levels than expected, indicating partial lethality [22]. Surviving *H3f3b*^*−/−*^ mice were infertile. Examination of embryos at E12.5 showed abnormal development and growth failure in the majority of *H3f3b*^*−/−*^ embryos [22].

Furthermore, MEFs isolated from *H3f3b*^*−/−*^ mice showed alterations in cell cycle dynamics compared to WT MEFs, and there was a significant increase in chromosomal bridges and abnormal chromosome numbers, suggesting genomic instability in the absence of *H3f3b* [22]. Staining experiments revealed that knockout MEFs also exhibited increased pericentric heterochromatin and kinetochore proteins, indicating centromere disfunction [22].

Microarray gene expression analysis comparing WT and *H3f3b*^*−/−*^ MEFs isolated from littermates at E12.5 showed surprisingly modest changes in overall gene expression as a result of *H3f3b* knockout [22]. Affected genes were enriched in categories related to histones, DNA synthesis, centromeres, and mitotic regulatory factors. At the protein level, H3S10P, H3K4me3, and H3K9ac levels were reduced [22].

Further studies were completed with these mice to investigate the observed fertility defects of *H3f3b* loss. Analysis of *H3f3b*^*−/−*^ testes revealed abnormal architecture in the seminiferous tubules, and a decrease in sperm concentration as well as sperm abnormalities including decreased motility, high levels of sperm with abnormal heads, and increased apoptosis in the tubules [21], further supporting the idea of an important role for H3.3 proteins in proper chromatin condensation and chromosome segregation. At the epigenetic level, H3K9me3 levels were higher in *H3f3b*^*−/−*^ testes compared to WT, while H3K4me3 levels were slightly decreased [21]. *H3f3b*^*−/−*^ testes also showed reduced Prm1 staining, indicating that *H3f3b* plays a role in the switch to protamine-based chromatin during spermatogenesis [21].

Another study employed a different Cre-LoxP approach to knockout *H3f3b*. H3f3b+/− mice could be generated through a standard Cre-approach, but heterozygous mice were found to be infertile and couldn’t be used to generate knockouts. To get around this, the researchers used a system where Cre is inserted into an X-linked gene (Hprt1), which results in excision of the floxed sequence shortly after fertilization of Cre-containing oocytes in females who are heterozygous for the Cre allele [23]. This study observed more severe phenotypes both in *H3f3b*^*+/−*^ and *H3f3b*^*−/−*^ compared to the previous study. Using this knockout approach, *H3f3b*^*+/−*^ mice were smaller than WT littermates at weaning, and while heterozygous *H3f3b*^*+/−*^ females were fertile, males were infertile [23], necessitating the alternative approach to study the effect of homozygous deletion of *H3f3b*. In approximately half of cases, development of *H3f3b*^*−/−*^ embryos did not proceed past the early post-implantation stage [23]. For those embryos that survived, about 24% died between E13 and E18.5, or showed large-scale abnormalities, and the rest died shortly after birth due to respiratory defects [23].

To examine the role of *H3f3b* in female gametogenesis, a Zp3-Cre line was used to delete *H3f3b* specifically in the follicle cells [23]. These females had dramatically reduced fertility compared to WT females. This was found to be caused by reduced fertilization of ova, as well as cleavage failure. In these cases, the zygotes did not accumulate high levels of H3Sph, which is a marker of late prophase [23]. When both *H3f3a* and *H3f3b* were both conditionally deleted, oocytes were found to be completely inviable [23].

As with *H3f3a* knockouts, differences in phenotypes obtained using different knockout approaches for *H3f3b* may be due to the timing of the knockout, the persistence of H3.3 protein coded by *H3f3b* for a long period of time after knockout, the background strain of the mice, or possibly the mechanism of disruption of the *H3f3b* locus itself.

## H3.3 double knockout

Surprisingly, another study using mice from mixed backgrounds (C57BL/6 and 129) and a Cre-based system to generate germline knockouts of *H3f3a* and *H3f3b* found single knockout mice for each gene to be normal and fertile [24]. When the two lines were crossed to generate double knockouts, the embryos usually did not survive past E6.5, suggesting either failure to implant, or to develop further after implantation. In examining combinations of knockout alleles, *H3f3a*^*−/−*^* H3f3b*^*+/−*^ mice died perinatally due to breathing problems. *H3f3a*^*+/−*^ *H3f3b*^*−/−*^ males and females both developed normally [24], however females were fertile while males were infertile due to loss of germ cells.

*Sox2-*conditional double knockout mice were generated to study the effects of H3.3 loss at a slightly later timepoint during development, as Cre expression starts during the blastocyst stage when under control of the *Sox2* promoter. Even with delayed onset of the knockout, *Sox2*-Cre H3.3 double knockout embryos show delayed growth and an increase in cell death at all timepoints and were reabsorbed by E10.5 [24]. These conditional knockout mice had significantly elevated p21 expression indicating activation of the p53 pathway as a likely cause of the observed cell death and cell cycle arrest phenotype in knockout embryos [24].

Interestingly, and in agreement with previous studies [22, 25], RNA-seq comparing WT and H3.3 knockout embryos revealed only modest effects of loss of H3.3 on gene expression; less than 5% of expressed genes were affected [24]. Among those genes most highly upregulated was the cell cycle inhibitor *Cdkn2a* [24]. Together, these results suggest that in a p53 WT background, mitotic defects caused by loss of H3.3 activate the p53 pathway, leading to cell cycle arrest and the observed growth defects.

More detailed analysis showed that H3K9me3 and H3K36me2 levels were reduced at telomeres, and heterochromatin domains were characterized by more open chromatin [24]. These differences could disrupt the normal functioning of centromeres and telomeres and thereby contribute to the observed genomic instability in H3.3 knockout mice and cells.

A recent paper studied the effect of *H3f3a* and *H3f3b* co-deletion specifically in developing neurons [19]. They knocked out *H3f3a* and *H3f3b* in neural progenitor cells using Emx1-Cre, and specifically in excitatory neurons after terminal mitosis using Neuro6d-Cre. In both situations, knockout mice were born alive but died within a few hours of birth [19]. These mice show changes to neuronal fate and identity as well as defects in axon projection and development. Transcriptomic and epigenomic analyses found that accumulation of H3.3 in chromatin is required for properly setting up the transcriptome in newly post-mitotic neurons and regulating the levels of H3K4me3 and H3K27me3 at these genes [19].

## H3.3 knockout in ESC

H3.3 double-depletion and double knockout strategies have also been used to study the roles of H3.3 in embryonic stem cells. Jang et al found that H3.3 double knockout ES cells undergo rapid cell death, primarily due to activation of the p53 pathway as a result of mitotic defects [24]. Due to the severe growth defect in these cells that made further characterization difficult, a p53 null mutation was crossed into the conditional knockout lines. With the addition of p53 knockout, the embryos survived until E11.5, and the growth defect was significantly diminished at all timepoints observed [24]. In cell culture, the p53 null background rescues H3.3 knockout cell growth phenotype, however the cells had significantly elevated levels of mitotic defects including lagging chromosomes and anaphase bridges [24]. Comparison of H3.3 knockout MEFs with or without p53 knockout yielded similar results: p53 knockout significantly increased the growth rate of H3.3 knockout MEFs, however these cells had higher levels of mitotic defects and elevated levels of H2A.X S139 phosphorylation, an indication of DNA damage [24].

Another study employed a zinc finger nuclease (ZFN) strategy to knockout both *H3f3b* alleles, and shRNA against *H3f3a* in mESC, resulting in strong depletion of total H3.3 in cells. These cells showed reduced nucleosome turnover at active and bivalent genes, and significant depletion of H3K27me3 at bivalent genes [25]. Consistent with mouse studies, RNA-seq analysis revealed only a small group of genes was affected by H3.3 depletion. Notably, they did not observe an effect of loss of the majority of H3.3 on stem cell self-renewal, proliferation, or proper chromosome segregation [25]. Another study with complete H3.3 null ESC found reduced levels of H3K27ac and other acetylation marks at distal enhancers, and determined this to be dependent on phosphorylation of H3.3 at Ser31, a residue unique to histone H3.3 [14].

## H3.3 mutations in human disease

Despite producing an identical protein, *H3F3A* and *H3F3B* mutations are uniquely associated with several different diseases. In humans, mutations in *H3F3A* but not *H3F3B* are strongly associated with pediatric high-grade gliomas, where approximately 70% of tumors bear a lysine to methionine (K27M) mutation in the sequence coding for the N-terminal tail of H3.3, and another 10–15% have a glycine to arginine or valine (G34R/V) mutation in *H3F3A* [29–31]. These mutations are associated with many gene expression and chromatin changes, as well as genome instability [30, 32]. One of the strongest effects of mutant *H3F3A* occurs at the level of chromatin structure and epigenetic regulation. Indeed, the H3.3K27M mutation is associated with sharp reductions in the repressive histone modification H3K27me3 and increases in the active modification H3K27ac, as well as the creation of spurious super enhancers at genes with cancer-promoting properties [33–35]. This pattern appears distinct from the situation in H3.3 knockout mice, where loss of H3.3 protein is only correlated with modest changes in histone modifications and gene expression [22].

It is interesting to note that genomic instability is a hallmark of both mutant H3.3 and knockout of H3.3 genes, highlighting important functions for H3.3 in maintaining chromatin structure, regulating chromosome segregation and promoting genome integrity throughout development.

H3.3 mutations are also associated with other human diseases: a K36M mutation specifically in *H3F3B* is observed in a subset of chondroblastomas, mutations of *H3F3A* are found in giant cell tumors of the bone, and overexpression of *H3F3A* in lung cancer leads to aberrant histone deposition and activation of metastasis-associated genes [36, 37].

At the same time, *H3f3a* and *H3f3b* do appear to share many functions. In one example, functionally similar or identical de novo germline mutations in either *H3F3A* or *H3F3B* have recently been identified in patients with a rare neurodegenerative disorder [38]. Patients with this condition suffer from developmental delay, epilepsy, neurodegeneration, and, in some cases, congenital abnormalities [38]. The mutations found in this condition are predicted to disrupt core H3.3 functions, including interactions with DNA, with other histones in the nucleosome, or with chaperone proteins, which may explain why mutations in *H3F3A* or *H3F3B* can lead to similar or even the same phenotypes [38]. When either gene is uniquely associated with a particular disease, it is possible that is explained by their unique expression patterns in distinct cells or the chromatin at either locus conferring more susceptibility to mutations.

## H3.3 depletion in other species

H3.3 loss of function has also been studied in other species. In *Caenorhabditis elegans* H3.3 is highly expressed and incorporated into chromatin throughout development and during adulthood, with the highest levels of H3.3 observed in post-mitotic cells. H3.3-deficient animals developed normally and are fertile, however they had lower survival rates when exposed to stress [39]. H3.3 loss in *Xenopus laevis* caused developmental defects during late gastrulation including spina bifida, open blastopore, and shortened anteroposterior axis [40].

In *Drosophila*, where H3.3 makes up approximately 25% of the total H3 protein in the cell, single H3.3 knockouts where phenotypically normal, but double knockouts were infertile and had reduced viability [41]. Examination of the testes of H3.3-double-deficient flies showed defects in chromosome condensation and segregation during meiosis, with lagging chromosomes frequently observed in Anaphase I and chromosome bridges in Anaphase II, suggesting a crucial role for H3.3 in chromosome organization and condensation during gamete production [41]. These defects can be rescued by ectopic expression of the canonical histone H3.2 [41], suggesting the total level of histone H3 could be more important than the specific variant being expressed in some contexts. The infertility and chromosome segregation defects observed in H3.3 deficient *Drosophila* are similar to defects seen in H3.3 knockout mice and suggest an evolutionarily conserved role for H3.3 in fertility and, more specifically, chromosome segregation during gamete production.

## H3.3 chaperone knockouts

Specific chaperone proteins are required for the incorporation of histones into chromatin. For example, the HIRA chaperone complex is responsible for depositing H3.3 into chromatin at active regions, while the ATRX/DAXX complex incorporates H3.3 at telomeres and pericentromeric heterochromatin. *Hira* knockout results in embryonic lethality around E10.5, with multiple major developmental abnormalities indicative of patterning defects originating during gastrulation [42].

Similar to H3.3, HIRA plays an important role in gamete generation. When *Hira* is specifically knocked-out during oogenesis using a Zp3-Cre system, oocytes develop normally, and females undergo ovulation, but are infertile [43]. Further examination indicated that *Hira* knockout in the oocyte causes zygotes to fail to reach the 2-cell stage, even if the sperm are from WT males [43]. Fitting with the observation that after fertilization the male pronucleus appears devoid of histones in *Hira* mutants, is the idea that H3.3 and its chaperone together have a unique role in histone deposition at this timepoint. Sperm DNA mostly has protamines rather than conventional histones, and needs to be repackaged after fertilization occurs [43]. Since this repackaging occurs before the first cell cycle, only a cell cycle-independent H3 variant in the form of H3.3 can be used for this process, and HIRA is a crucial part of the H3.3 deposition pathway.

Loss of function of the ATRX/DAXX chaperone complex, which is responsible for H3.3 deposition at heterochromatic and pericentromeric regions, results in a number of defects. *Atrx* knockout mESC cell lines have increased levels of DNA damage compared to WT mESC, and delayed progression through S phase, due to replication fork stalling at repetitive regions and sites of heterochromatin, where ATRX is known to bind [44]. Similar S phase delay, DNA damage, and telomere fragility are also observed in *Atrx* conditional knockout in myoblasts [45] and neural progenitor cells [46]. *Daxx* knockout also results in early embryonic lethality, around day E9.5. *Daxx*^*−/−*^ embryos are smaller than WT littermates and also appear highly disorganized [47]. Cells isolated from these mice show increased rates of apoptosis compared to WT controls, possibly due to defects in DNA damage response or other related pathways [47].

## Knockout of histone variants with functions related to H3.3

There is some general overlap in knockout phenotypes for variants H2AZ and H2AX with H3.3 and ATRX knockouts. For example, H2AX knockout mice are born at expected ratios, but have a growth defect compared to their WT littermates [48]. This phenotype is at least partially attributable to cell cycle arrest, due to the key role of H2AX phosphorylation in the DNA damage response [48]. Like H3.3, H2AX plays an important role in genome stability, highlighted by the finding that H2AX^−/−^ MEFs have a significant increase in karyotypic abnormalities including chromatid breaks and dicentric chromosomes. H2AX^−/−^ female mice are sub-fertile, while H2AX^−/−^ male mice are completely infertile, with thinner seminiferous tubules and no detectable mature sperm [48].

Histone H2AZ is another variant of histone H2 that shares some similarities with H3.3. It is also encoded by 2 genes (*H2az.1* and *H2az.2*) although lacking introns. H2AZ is expressed throughout the cell cycle, and is enriched at euchromatic regions as well as at heterochromatin and centromeres. Indeed, H2AZ and H3.3 are often found together in “double variant” nucleosomes that have higher levels of instability, contributing to high nucleosome turnover and open chromatin in these regions [49]. H2AZ knockout mice die early in development [50], and siRNA knockdown of H2AZ results in major chromosomal abnormalities including lagging chromosomes and the presence of chromatin bridges between dividing nuclei [51]. These effects are at least in part due to the disruption of HP1α binding at heterochromatin [51]. The overlap in fertility, growth, and chromosome segregation phenotypes between different histone variant knockouts underscores the importance of histone variants in proper chromosome segregation during processes like gametogenesis, as well as the many rounds of mitosis required for proper growth and development of an organism.

## Conclusions

While knockout and depletion studies on histones can be challenging due to the many histone genes and proteins, the presence of variants, and the long half-lives of these proteins, a number of recent papers have successfully employed these strategies to better understand the roles of H3.3. These have identified key roles for histone H3.3 in genome integrity, including proper segregation of chromosomes during cell division, and crucial and non-redundant roles of *H3f3a* and *H3f3b* in gamete formation in mice, highlighting the important and unique regulatory roles of histone variants like H3.3 (Fig. 1). The relatively mild effects of loss of H3.3 on the transcriptome have been consistently observed, suggesting either a lesser role in gene expression or complex redundancies or compensatory mechanisms. Overall, H3.3 knockout studies provide important context for understanding the mechanisms of H3.3 mutations in cancers and brain development, and may uncover roles for H3.3 in additional tissues and diseases in the future.

## Data Availability

Not applicable.

## Abbreviations

WT: Wild type
MEF: Mouse embryonic fibroblast
